# Response to letter to the editor regarding: ‘osteoporosis screening and major osteoporotic fracture prediction by cranial computed tomography-derived Hounsfield units: a multi-centre study on opportunistic osteoporosis screening’

**DOI:** 10.1080/07853890.2025.2580715

**Published:** 2025-10-30

**Authors:** Robert Wakolbinger-Habel, Daniel Arian Kraus

**Affiliations:** ^a^ Department of Physical and Rehabilitation Medicine (PRM), Vienna Healthcare Group, Clinic Donaustadt, Vienna, Austria; ^b^ Division of Endocrinology and Diabetology, Department of Internal Medicine, Medical University of Graz, Graz, Austria

Dear Editor

We are delighted to receive a forward-looking comment on our study.

We concur with Song et al. [1] that further research for implementation of opportunistic osteoporosis screening by CCT-derived HU into clinical practice focusing on various age groups as well as validation of standardized protocols for different CT scanners is of crucial importance.

A promising application for minimizing clinical workload and inter-rater variability for region of interest placement is the use of artificial intelligence (AI). Especially as the impression of utilization of AI in the context of opportunistic osteoporosis screening to decrease the treatment gap of fragility fractures is rising.

In this context, we already published preliminary results indicating the potential of AI in vertebral fracture detection. We reported a sensitivity of 89.5% and specificity of 98.0% for identification of higher-grade vertebral fractures (Genant 2/3) in CT studies of the chest and abdomen by the prototype of a deep learning AI [2].

Opportunistic osteoporosis screening by CCT derived HU may have a unique selling point as especially elderly patients receive this radiological examination in clinical routing (e.g. to rule out intracranial bleeding). Furthermore, the frontal bone might not be so prone to degenerative changes compared to vertebrae-based HU measurements.

By CCT-based opportunistic osteoporosis screening, a major contribution to prevention of osteoporotic fractures as well as the reduction of treatment gap in patients with sustained fragility fractures could be achieved. Incorporation of AI to standardize and automatize workflows may increase usability in clinical practice. This could also affect public health by potentially decreasing osteoporosis-associated costs, morbidity, mortality and increasing quality of life. However, the integration of AI based approaches into clinical practice still requires future validation studies.

## Data Availability

Data sharing is not applicable to this article as no data were created or analysed.
